# Skin biopsy in adult-onset Still’s disease: A specific, rapid, and widely available diagnostic test

**DOI:** 10.70962/jhi.20260088

**Published:** 2026-09-18

**Authors:** Derrick H.Y. Chong, Caroline Spaner, Ibrahim Elsharawi, Mark Trinder, Haya Al Bitar, Mariam Goubran, Amelia Perrotta, Rachael Horner, Dilys Chen, Jennifer Corpuz, Kun Huang, Trudy Taylor, Ian Marie Lano, Kerri Purdy, Jan P. Dutz, Sylvia Pasternak, Richard I. Crawford, Luke Y.C. Chen

**Affiliations:** 1 Department of Medicine, University of Toronto, Toronto, Canada; 2 Division of Hematology, University of Toronto, Toronto, Canada; 3 Department of Pathology, Dalhousie University, Halifax, Canada; 4 Department of Pathology and Laboratory Medicine, University of British Columbia, Vancouver, Canada; 5 Faculty of Medicine, Dalhousie University, Halifax, Canada; 6 Division of Hematology, University of British Columbia, Vancouver, Canada; 7 Division of Dermatology, Dalhousie University, Halifax, Canada; 8 Division of Rheumatology, University of British Columbia, Vancouver, Canada; 9 Division of Rheumatology, Dalhousie University, Halifax, Canada; 10 Division of Dermatology & Skin Science, University of British Columbia, Vancouver, Canada; 11 Division of Hematology, Dalhousie University, Halifax, Canada; 12 Coastal Program for Rare Inflammatory Diseases, Nova Scotia Health Research & Innovation Hub, Halifax, Canada

## Abstract

Adult-onset Still’s disease (AOSD) is a diagnostic challenge, as its features overlap with many inflammatory syndromes and no single finding is diagnostic. Skin biopsies showing dyskeratotic keratinocytes (DKs) in the superficial epidermis, including the horny layer, are thought to be highly specific for AOSD but are not included in current diagnostic or classification criteria. We conducted a retrospective clinicopathological study of 16 adults with confirmed AOSD and skin biopsies from a Canadian rare diseases program, and developed a differential diagnosis for superficial DKs using expert consensus and literature review. Clinical morphology varied, but histopathology was similar: 12 of 16 patients had DKs or necrotic keratinocytes in the upper epidermis. Superficial DKs have been reported in conditions distinct from AOSD, but not in its major mimics. Skin biopsy is low-cost, minimally invasive, and widely available and should be performed early in suspected AOSD; superficial DKs should be added to future diagnostic and classification criteria.

## Teaser

AOSD is challenging to diagnose. Chong et al. examine the clinical utility of skin biopsy, an inexpensive and widely available test, in 16 patients with AOSD. They demonstrate that dyskeratotic keratinocytes in the superficial layers are useful for diagnosing AOSD.

## Introduction

Adult-onset Still’s disease (AOSD) is a rare autoinflammatory disease that classically presents in patients older than 16 years with fever >39°C, an evanescent salmon-colored maculopapular rash, polyarthritis, myalgia, lymphadenopathy, pharyngitis, and elevated acute-phase reactants such as ferritin and C-reactive protein (CRP) (1, 2). AOSD is considered on a spectrum with its pediatric counterpart, systemic juvenile idiopathic arthritis (JIA), and both fall under the umbrella term Still’s disease (3, 4). A subset has a severe, or catastrophic, form of the disease (catastrophic AOSD [cAOSD]) characterized by critical illness and severe end-organ damage (5, 6, 7, 8).

Delayed diagnosis is a significant clinical problem in AOSD, owing in part to a lack of specific biomarkers and overlap of key features with other inflammatory syndromes such as hemophagocytic lymphohistiocytosis (HLH) and sepsis (4, 9, 10, 11). The Yamaguchi (12) and Fautrel (13) classification criteria are most commonly used for diagnosis in clinical practice and are synthesized in the most recent European Alliance of Associations for Rheumatology (EULAR)/Paediatric Rheumatology European Society (PReS) recommendations (Table 1) (4). However, patients may take weeks or even months to fulfill these criteria. One study reported a median delay of 4 mo from presentation to diagnosis of AOSD (14). Patients with severe Still’s or cAOSD often do not have the classic evanescent skin rash or arthritis, and thus, despite greater urgency for diagnosis in those cases, recognition may be delayed.

**Table 1.** Selected diagnostic/classification criteria for diagnosis of AOSD

Yamaguchi (12) (classification—1992)	Fautrel (13) (classification—2002)	Cush (2) (diagnostic—1987)	EULAR/PReS (4) (expert consensus diagnostic recommendations)
≥5 criteria (≥2 major)	≥4 major or 3 major + 2 minor	AOSD diagnosis by Yamaguchi or Fautrel plus ≥1 ICU-level organ failure:	Operational definitions to facilitate early diagnosis (no formal scoring system)
**Major criteria** Fever >39°C for ≥1 wk Arthralgia/arthritis for ≥2 wk Typical rash Leukocytosis >10 × 10^9^/liter with >80% PMN cells **Minor criteria** Sore throat Lymphadenopathy Hepatosplenomegaly Abnormal LFTs Negative ANA and RF	**Major criteria** Fever ≥39°C Arthralgia Transient erythema Pharyngitis PMN cells ≥80% Glycosylated ferritin ≤20% **Minor criteria** Maculopapular rash Leukocytosis ≥10 × 10^9^/liter	Acute respiratory failure Lung infiltrate Pleural effusion Cardiovascular failure (shock, myocarditis, and tamponade) Hematologic disorder (DIC, hemophagocytosis, and thrombotic microangiopathy)	Spiking fever ≥39°C for ≥7 days Evanescent, salmon-pink, truncal rash associated with fever spikes; atypical rashes possible Arthralgia/myalgia; arthritis may develop later (supportive but not required) Neutrophilic leukocytosis Elevated CRP and ferritin Elevated IL-18 and/or S100 proteins strongly supportive when available
Exclusion of infection, malignancy, and other rheumatic disease	Exclude infection and malignancy	Exclusion of alternative causes (infection, drug reaction, or malignancy)	Exclusion of infection, malignancy, other immune-mediated inflammatory diseases, and monogenic autoinflammatory disorders

ANA, antinuclear antibody; RF, rheumatoid factor; PMN, polymorphonuclear; ICU, intensive care unit; DIC, disseminated intravascular coagulation; LFT, liver function tests.

Clinical cutaneous findings are central to diagnosis in AOSD. The “typical rash,” defined as a major diagnostic criterion by Yamaguchi, is a macular or maculopapular nonpruritic salmon-pink eruption appearing during fever and fading when the patient is afebrile. However, physical exam findings are heterogeneous and may fluctuate over the course of a patient’s illness. The classic evanescent, bright-pink rash of the Yamaguchi criteria may be subtle, transient, or difficult to recognize, particularly in darker skin types. Moreover, “atypical rashes” are quite common. Many patients with AOSD may have urticarial, papular, linear, or lichenoid morphologies, and these may be persistent rather than transient (15, 16, 17, 18, 19). Atypical morphologies can also coexist with classical features in the same patient (16).

While all existing classification criteria include clinical findings of rash, none include skin biopsy findings as a criterion. Biopsy of patients with the “typical” evanescent rash is rarely done, as this typically demonstrates nonspecific lymphocytic and neutrophilic infiltrates, as well as heterogeneous findings as described by both Lee et al. and Larson et al. in a study of 14 biopsy specimens (16, 20). In 2005, the seminal paper by Lee et al. described the presence of dyskeratotic keratinocytes (DKs) confined to the upper epidermis, associated with persistent pruritic papules in AOSD; they describe 11 patients with AOSD who had persistent eruptions, nine of which also had evanescent rashes (21). The defining histopathologic feature of the persistent pruritic eruptions (PPE) was DKs in the upper epidermis, specifically within the horny layer, with a lymphocytic and neutrophilic infiltrate (21). This distinct finding of DKs in the upper epidermis, and mostly in the horny layer, has since been described in numerous studies (15, 16, 17, 18, 19, 20, 22, 23, 24, 25, 26, 27, 28, 29, 30, 31). This histological finding, and specifically the lack of DKs present in the deeper epidermis, in the correct clinical context, is thought to be quite specific for AOSD, suggesting that skin biopsy may be a useful and underappreciated diagnostic test.

In this study, we examine a series of patients cared for in a Canadian rare disease program with confirmed AOSD who had a skin biopsy done. Many of these patients were referred because they were a diagnostic conundrum. We then conducted a review of the literature on other conditions in which DKs are described in skin biopsy. Our aim was to evaluate the potential role of cutaneous histopathology, and specifically the finding of DKs in superficial epidermis, in the diagnosis of AOSD.

## Results

### Patient demographics and clinical features

16 patients were included in the study. Patient demographics and laboratory values at diagnosis are summarized in Table S1. All patients presented with fever. Common systemic findings included arthralgias/polyarthritis in all patients, pharyngitis (*n* = 11), and splenomegaly/hepatomegaly (*n* = 3). Four patients had cAOSD, including severe complications such as macrophage activation syndrome (MAS), disseminated intravascular coagulation (DIC), or myocarditis requiring intensive care unit (ICU) management (6).

### Cutaneous findings

**Figure S1.**
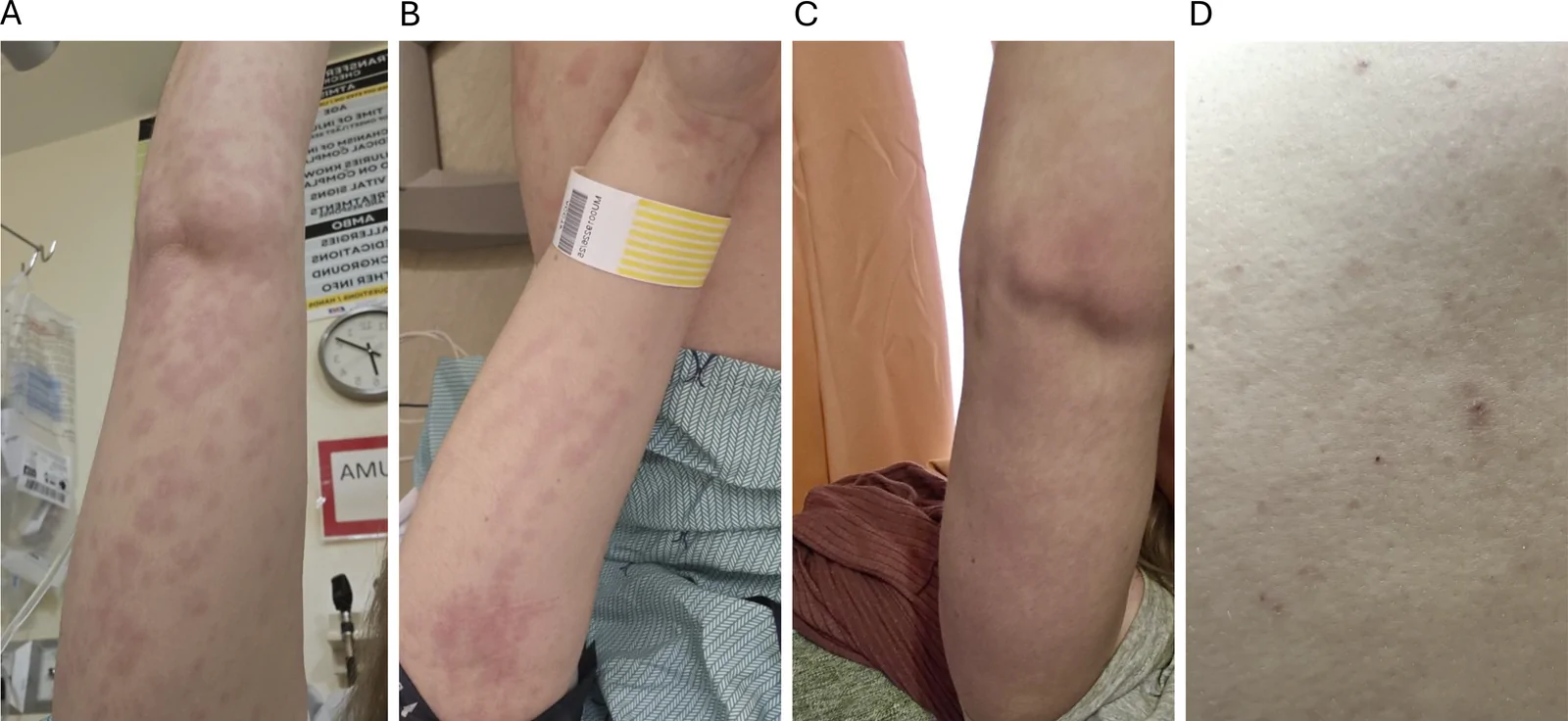
**Some examples of the clinical skin lesions found in patients in this study are provided. (A and B) **Examples of clinical skin lesions (case 16) (A: right arm, B: left arm). Initial presentation of erythematous, urticated papules and plaques with suspected Koebner phenomenon associated with fever and systemic symptoms.** (C) **Right arm recurrence of erythema accompanied by arthralgias and intense pruritus on tapering of oral corticosteroids.** (D) **Upper back area of skin biopsy with few scattered excoriated papules where erythema and gross cutaneous inflammation had largely resolved; biopsy taken of non-excoriated, perilesional skin.

Morphological descriptions of the skin lesions, with associated histopathology findings, are summarized in Table 2. PPEs were found in cases 4, 15, and 16, while only case 7 described a classic salmon-colored evanescent rash (Table 2). Charts included descriptions of primary lesions, including only flat or macular (cases 2, 9, 12, and 13) lesions, maculopapular (cases 1, 3, 7, 10, 11, and 15), and erythema (cases 6, 8, 14, and 16). Some examples of clinical lesions are in Fig. S1. In most cases, comments on pruritus, evanescence, or persistence were not found. DKs in the epidermis were found in PPE lesions (cases 4 and 16), macular lesions (cases 2, 9, 12), maculopapular lesions (cases 3, 7, 10, and 11), and cases without a primary lesion (cases 6 and 8). Notably, dyskeratosis was not found in one case describing macular lesions (case 13) and two cases describing maculopapular lesions (cases 1 and 15).

**Table 2.** Morphological description of skin rash and histopathologic findings in AOSD

Case (DK)	Description	Key skin biopsy findings	Clinicopathologic Correlation
1 (NO)	Full-body maculopapular rash, predominantly non-pruritic	Superficial perivascular lymphocytic dermatitis	Viral exanthem, morbilliform drug eruption
2 (YES)	Extensive blanchable erythematous macules coalescing into patches involving the arms, legs, and torso	Upper-epidermal dyskeratosis with sparse superficial perivascular lymphocytic infiltrate	PPE of AOSD
3 (YES)	Blanchable, non-pruritic maculopapular rash involving the upper torso, arms, and lateral ankles	Upper-epidermal necrotic keratinocytes with superficial mid-dermal perivascular lymphohistiocytic infiltrate; mild dermal mucin	PPE of AOSD
4 (YES)	Persistent pruritic linear plaques of the trunk and buttocks	Upper-epidermal dyskeratosis with mild perivascular lymphoplasmacytic infiltrate and occasional neutrophils	PPE of AOSD
5 (YES)	Linear hyperkeratotic plaques with areas of hyperpigmentation involving the upper and lower back and gluteal region	Epidermal dyskeratosis with parakeratosis and spongiosis; mixed perivascular inflammatory infiltrate in the mid-upper dermis	PPE of AOSD
6 (YES)	Diffuse erythematous rash involving the abdomen, arms, and legs	Sparse epidermal necrotic keratinocytes with focal parakeratosis; prominent superficial and deep perivascular/interstitial eosinophilic infiltrate	PPE of AOSD
7 (YES)	Raised, salmon-colored, blanchable maculopapular rash involving the distal extremities	Sparse epidermal necrotic keratinocytes with superficial perivascular and interstitial neutrophils	PPE of AOSD
8 (YES)	Blotchy erythematous rash involving the back, neck, abdomen, and legs	Upper-epidermal dyskeratosis with focal subcorneal pustules	PPE of AOSD
9 (YES)	Hyperpigmented macular rash involving the anterior chest, neck, and left inguinal region	Upper-epidermal dyskeratosis with parakeratosis and mild spongiosis; superficial perivascular lymphocytic infiltrate with occasional eosinophils	PPE of AOSD
10 (YES)	Widespread maculopapular, blanchable coalescing lesions along the arms, legs, and torso	Upper-epidermal necrotic/DKs, mild spongiosis, subtle interface vacuolar changes, and mid-dermal perivascular lymphocytic infiltrate with scattered neutrophils	PPE of AOSD
11 (YES)	Widespread, symmetric erythematous papules and macules coalescing into patches involving the trunk and extremities	Upper-epidermal dyskeratosis with sparse superficial perivascular infiltrate and focal vacuolar interface change	PPE of AOSD
12 (YES)	Well-demarcated, flat, and blanchable erythematous lesions on forearms and coalescing to involve most of the body	Upper-epidermal necrotic keratinocytes; parakeratosis; mixed superficial and deep perivascular inflammatory infiltrate of plasma cells and eosinophils	PPE of AOSD
13 (NO)	Migratory, blanchable, non-raised erythematous rash; occasionally pruritic	Nonspecific superficial perivascular and interstitial neutrophilic/lymphocytic infiltrate, mild hyperkeratosis	Classic evanescent AOSD rash, urticaria
14 (NO)	Migratory, intermittent blanchable erythematous rash involving the upper body	Superficial perivascular and interstitial mixed inflammatory infiltrate (neutrophils and lymphocytes), mild hyperkeratosis, papillary dermal edema	Classic evanescent AOSD rash, urticaria
15 (NO)	Pruritic light pink macules and papules with erythematous plaques on trunk/extremities	Light superficial perivascular lymphocytic infiltrate with occasional interstitial neutrophils	Viral exanthem, morbilliform drug eruption
16 (YES)	Pruritic erythematous urticated plaques on torso and extremities; largely resolved at time of biopsy with scattered excoriated papules and minimal residual erythema	Occasional DKs in the mid-to-upper epidermis with otherwise unremarkable epidermis and minimal dermal inflammation	PPE of AOSD

DK, dyskeratotic keratinocytes present in superficial layers (YES) or not (NO).

**Figure 1.**
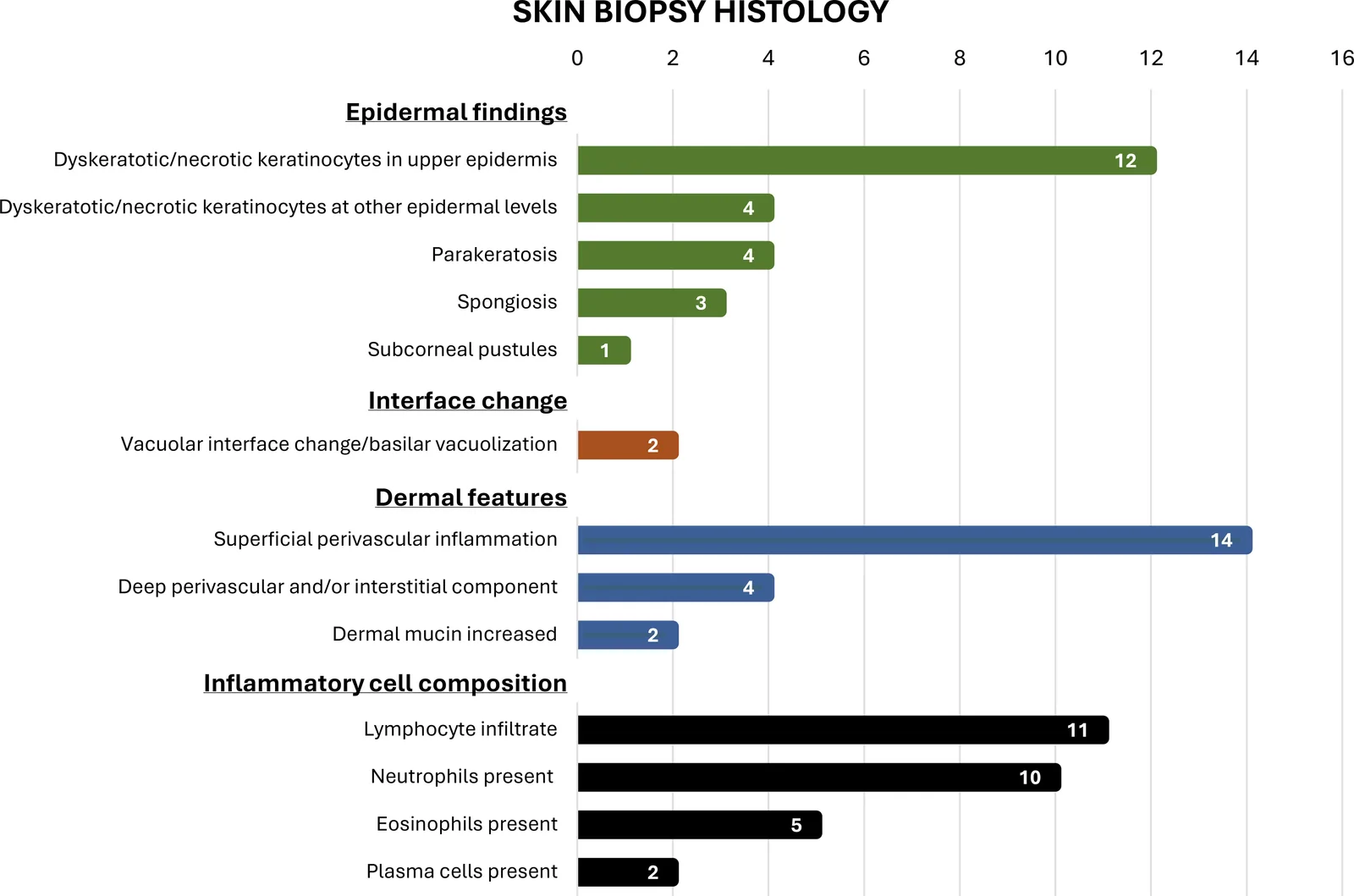
Summary of skin biopsy histopathologic features in AOSD.

**Figure 2.**
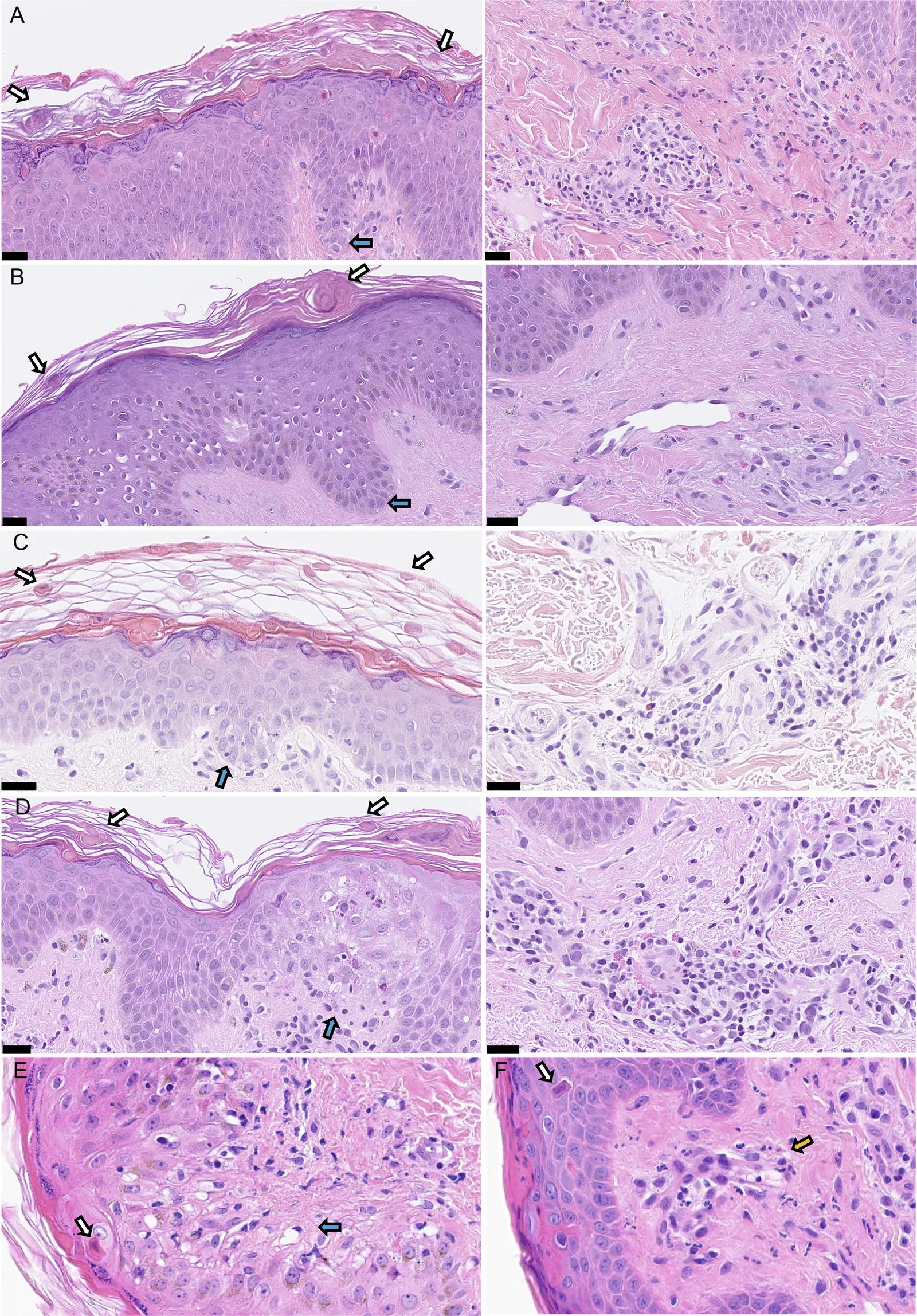
**Histological features of skin biopsy in patients with Still's disease. (A–D) **Spectrum of histopathological changes in skin biopsy specimens of patients with AOSD (hematoxylin and eosin stain). **(A–D) **Panels represent four unique patients from one center: Sections show DKs in the stratum corneum of the epidermis (white arrows), focal vacuolar interface change (blue arrows), and scattered perivascular mixed inflammation composed of lymphocytes, eosinophils, and neutrophils in the superficial dermis. The scale bars each represent 25 μm. **(E and F)** Panels demonstrate medium-power-view (×200) findings noted in two different patients from another center. E demonstrates prominent vacuolar interface (blue arrow), and F shows a predominantly neutrophilic pattern of superficial and perivascular inflammation (yellow arrow). Both cases show superficial DKs, as noted with the white arrows.

Skin histopathology findings are summarized in Fig. 1. DKs were found in the superficial layers in 12 of 16 patients. Regarding the composition of the inflammatory infiltrate, our findings were generally concordant with those previously reported by Kim et al. (32), with many cases exhibiting a lymphocytic infiltrate and only two cases demonstrating plasma cells (Fig. 1). Notably, our study identified a relatively higher frequency of neutrophilic infiltrates (62.5% compared with 26.9% in Kim et al.) as well as eosinophilic infiltrates (31.25% compared with 3.8% in Kim et al.) (32). Perivascular inflammatory infiltrate in the upper dermis was prevalent, predominantly lymphocytes. Representative histology is shown in Fig. 2.

### Differential diagnoses of DKs

The literature review revealed that DKs have been reported in the superficial epidermal layers in at least seven conditions other than Still’s (15, 16, 17, 18, 19, 20, 22, 23, 24, 25, 26, 27, 28, 29, 30): acute graft-versus-host disease (aGVHD) (33, 34), toxic shock syndrome (TSS) (35), acute inflammatory erythema (sunburn) (36), phototoxicity (37), prurigo pigmentosa (38), irritant contact dermatitis (39), and verrucous phase of incontinentia pigmenti (40) (Table 3).

**Table 3.** Clinical differential diagnosis for singly dispersed necrotic keratinocytes in the superficial epidermis and cornified layers

Condition	Key non-cutaneous clinical features	Typical cutaneous findings	Other histological findings
**Systemic inflammatory disease**			
AOSD (1, 4, 13, 15, 16, 17)	High fever, myalgias, hyperferritinemia, and pharyngitis	Evanescent salmon-coloured eruption Linear urticaria Persistent pruritic papular eruption	Vacuolar interface dermatitis; mild dermal infiltrate of eosinophils/neutrophils
aGVHD (33, 34)	History of transplant, gastrointestinal symptoms	Erythematous macules and papules with varying body surface area, rarely bullae or erythroderma	Confluent or diffuse keratinocyte necrosis; variable superficial inflammatory infiltrates without neutrophils; vacuolar interface dermatitis with hair follicle involvement
**Bacterial-toxin-associated–mediated erythemas**			
TSS (35)	Recent surgery, high fever, myalgias, pharyngitis, and shock	Erythroderma or flexure-predominant macular eruption Desquamation of palms and soles	Variable dermal perivascular inflammatory infiltrate; epidermal spongiosis without interface dermatitis
**Photodermatologic disorders**			
Acute inflammatory erythema (sunburn) (36)	Exposure history	Erythema of sun-exposed skin with burning sensation and possible edema or blistering	Minimal dermal inflammation without eosinophils or vacuolar interface dermatitis
Phototoxicity (37)	Exposure to phototoxic agent and UV radiation	Erythema of sun-exposed skin with burning sensation and possible edema or blistering	Variable spongiosis and/or interface dermatitis; variable dermal inflammatory infiltrate
**Other inflammatory skin diseases**			
Early acute phase of prurigo pigmentosa (38)	Young adult female, systemically well except for ketosis	Pruritic erythematous papules and vesicles symmetrically distributed on the back, neck, and/or chest evolving into a reticulated configuration of hyperpigmented macules	Spongiotic dermatitis with intraepidermal and superficial perivascular lymphocytes; possible confluent keratinocyte necrosis and/or intraepidermal neutrophilic microabscesses; abundant bacteria within hair follicles
Irritant contact dermatitis (39)	History of exposure	Painful and/or pruritic erythema, edema and/or bullae on exposed sites	Spongiotic dermatitis with intraepidermal and superficial perivascular lymphocytes and neutrophils
**Genodermatoses**			
Verrucous phase of incontinentia pigmenti (40)	Family history, pediatric onset, dental, hair (“woolly”), nail, central nervous system, eye, and breast abnormalities	Erythematous, vesicular or verrucous linear plaques following lines of Blaschko	Eosinophilic spongiosis in earlier lesions; verrucous epidermal hyperplasia in later lesions; dermal lymphocytes, eosinophils, and/or melanophages, without neutrophils

## Discussion

Better diagnostic tests are urgently needed for AOSD, particularly for patients with severe or catastrophic disease. Unlike monogenic autoinflammatory disorders such as deficiency of adenosine deaminase 2 or vacuoles, E1 enzyme linked, X-chromosome, autoinflammatory, somatic (VEXAS) syndrome, genetic testing is generally not helpful in the diagnosis of AOSD (41). Imaging is rarely helpful for diagnosis. Several serum biomarkers have been proposed for AOSD. The Fautrel criteria (2002) include glycosylated ferritin <20% (13), and the recent EULAR/PReS (2024) criteria encourage the measurement of serum IL-18 and S100 proteins (4). Other candidate biomarkers include inflammatory cytokines (e.g., IL-1β, IL-6, TNF, and IFN-γ) and chemokines (CXCL9, -10, and -13) that drive autoinflammation in Still’s (10, 42). Our group has previously shown that sCD25 (also known as soluble IL-2, α chain) in combination with CRP and ferritin can be quite helpful in distinguishing AOSD from HLH (43). While all of these novel biomarkers have merit and should be studied and clinically applied where available, they share significant limitations:

(1) They are not widely available, particularly in lower-income jurisdictions.
(2) Even when available, they are often run as research tests rather than clinically validated assays.
(3) Turnaround times are often several days to weeks.
(4) They have not been extensively tested in other diseases, and thus specificity is not known. For example, a recent study has shown that severe COVID-19 is also associated with low glycosylated ferritin (44).
(5) The degree of elevation is not well defined. While sCD25 is “high” with respect to healthy controls in diseases like Still’s and thrombocytopenia, anasarca, fever/fibrosis, renal dysfunction, organomegaly (TAFRO) syndrome (43, 45, 46), it is only minimally elevated in those disease states compared with other disease states such as HLH, which have more exuberant T cell activation.

In contrast to the shortcomings of novel serum biomarkers, skin biopsy is a diagnostic test that is nearly universally available in every jurisdiction (47). In most centers, a punch biopsy can be interpreted within one working day of collection, and identification of DKs is straightforward for pathologists.

Our study confirms previous literature showing that DKs are a common finding in patients with AOSD and skin biopsies (16). Historically, two cutaneous eruption patterns are recognized in AOSD: the classic evanescent eruption and the PPE; the histological finding of DKs is traditionally associated with the latter (29, 48). A recent study of 518 patients shows that the clinical cutaneous manifestations of AOSD are more heterogeneous than previously thought (49). The classic evanescent rash was present in only 64%, and the remainder included macules (7.7%), urticaria (5.9%), erythema (5.2%), PPE (4.8%), and other findings. Although limited to retrospective clinical documentation, our study describes similar diverse cutaneous findings in AOSD and, surprisingly, the presence of DKs in the upper epidermis was found in patients where the clinical documentation did not always tend to describe PPE lesions. We find it unlikely that non-PPE lesions would have DKs in the upper epidermis, as literature for decades has largely only documented this in PPE lesions. Although a brief abstract by Shipp et al. described DKs in the upper epidermis with a morphological description of an evanescent morbilliform rash in AOSD, this was complicated by the further description of scaly papules present on the upper chest and extremities, which is not a classic AOSD rash (50). This further highlights the limitations of this method of study. Furthermore, our rare disease program tends to receive referrals for severe complex cases, and 25% of our patients in this study had catastrophic disease. In the study by Ruscitti et al. they identified 89/597 (15%) patients with life-threatening AOSD. Whether cAOSD tends to have atypical skin eruptions with DKs will also need to be studied further prospectively. Maeda-Ayoma et al. found that serum IL-18 levels were significantly higher in AOSD patients with DKs in skin biopsy compared with those without, suggesting that the DKs are associated with systemic autoinflammation rather than the clinical lesion morphology (29).

Our expert consensus and literature review show that the differential diagnoses associated with DKs in the upper layers of the epidermis are relatively specific and associated with niche clinical contexts. As summarized in Table 3, DKs explicitly found in the superficial epidermis and cornified layers are seen in at least seven other disease states, all of which are quite simple to distinguish from AOSD. However, Wolgamot et al. describe DKs in many settings, including lichenoid dermatoses such as lichen planus, erythema multiforme, lichen striatus, lichenoid drug eruptions, and GVHD; genodermatoses such as Hailey–Hailey disease, Darier disease, and incontinentia pigmenti; infections including HPV and herpes; autoimmune disease such as paraneoplastic pemphigus; and neoplasms including squamous cell carcinoma and adnexal tumors (31). Table 3 does not include secondary dyskeratosis. Furthermore, in diseases where DKs can present atypically, such as hand-foot-mouth disease, measles, thymoma-associated multiorgan autoimmunity, pityriasis lichenoides et varioliformis acuta, and nutritional deficiency, have not been included in the Table 3. A history and physical can often rule out diagnoses requiring exposures such as photodermatologic disorders or irritant contact dermatitis. Inflammatory lesions that may appear like persistent papules may be prurigo pigmentosa; however, patients are generally well with no systemic symptoms. aGVDH and TSS are both severe inflammatory responses with cutaneous findings that may clinically overlap with AOSD and have DKs in the upper epidermis. Consideration of aGVHD in an acutely ill patient with rash would be a priority in transplant patients. TSS is a consideration for those who have risk factors for *Staphylococcal* or *Streptococcal* infection and classically would also present with desquamation of the palms and soles. Given the limited differential diagnosis for the presence of DKs in the upper layers of the epidermis, it would be reasonable to perform a skin biopsy in a patient with an undifferentiated inflammatory diagnosis that may include AOSD.

Important mimics of severe AOSD and vice versa include vasculitis, sepsis, HLH, TAFRO syndrome, and other autoinflammatory syndromes such as VEXAS (51, 52). Cutaneous findings in vasculitis are of course quite distinct from AOSD (53). To our knowledge, DKs have not been reported in sepsis, non-AOSD-related HLH, TAFRO, or VEXAS (51, 54, 55, 56). While AOSD can certainly evolve into secondary HLH, in most cases it is a distinct autoinflammatory disease driven by IL-1, IL-6, and IL-18 and best treated with specific cytokine inhibitors, whereas HLH is driven by IFN-γ and T cell/macrophage activation and treated with cytotoxic chemotherapy (etoposide), corticosteroids, and JAK inhibition (8, 52). Moreover, patients with hyperferritinemic inflammation often undergo skin biopsy to rule out entities such as intravascular lymphoma. Pathologists receiving such specimens should comment not only on the presence or absence of malignancy but also on whether DKs are present.

None of the current diagnostic or classification criteria for AOSD include skin biopsy, or indeed any histological findings. Moreover, knowledge about cutaneous histology seems largely limited to the pathology/dermatopathology community, as clinical reviews that do mention skin biopsy often omit the significance of dyskeratotic or necrotic keratinocytes in superficial layers (42, 57). Failure to utilize readily available and clinically useful histological findings in AOSD stands in contrast with other autoinflammatory and cytokine storm syndromes. For example, abundant vacuoles in bone marrow erythroid and myeloid precursors are essential for recognition and diagnosis of VEXAS (58); hemophagocytosis is a sensitive but nonspecific finding in HLH, and patients with TAFRO typically have hyaline-vascular Castleman features in lymph nodes (Table S2). Since skin biopsy is a simple, widely available test that can potentially offer specific diagnostic information in AOSD, it should be included in future diagnostic and classification criteria.

The strengths of this study include the synthesis of differential diagnoses for DKs confined to the upper epidermis, as well as the availability of detailed biopsy reports and corresponding clinical manifestations for patients with AOSD. Our findings corroborate previously reported cutaneous histopathological findings of AOSD in the literature, present the knowledge gaps in heterogeneous cutaneous presentations in AOSD, and highlight the specificity of DKs to niche clinical situations.

Limitations of the study include the small sample size, lack of clinical images, and variable morphological descriptions by numerous clinicians. Given that the literature predominantly outlines persistent pruritic plaques to be the clinical finding with DKs in the upper epidermis, further studies would benefit from a larger cohort and available representative clinical images to ensure consistent histopathological correlation. Prospective studies designed with standardized dermatologic examination with explicitly systematic morphologic descriptions will be needed to determine whether this histopathologic pattern truly occurs independently of PPE-like morphology. This will be further complicated by the likely evolutionary nature of AOSD rash morphology, as a lesion appearing as a PPE may change into a different morphology over time. This list of other conditions included in the differential diagnosis of DKs in superficial layers requires some degree of expert pathological judgement; for example, some might include toxic epidermal necrolysis/Stevens-Johnson syndrome (TEN/SJS) in Table 3; however, in the opinion of the experts in the present study, TEN/SJS are more classically associated with necrotic keratinocytes in which the nucleus disappears completely (karyolysis), while DKs (at least before they reach the stratum corneum) maintain a viable nucleus.

This study suggests several areas for future research. The first is a comparison of cutaneous histology between AOSD, which is thought to be largely driven by IL-18, and other cytokine storm syndromes such as COVID-19, wherein diverse cutaneous lesions are driven by cGAS-STING–mediated IFN responses, and Schnitzler syndrome, an IL-1 inhibitor–responsive autoinflammatory disease that typically shows neutrophilic urticaria on skin biopsy (59). Second, while some cases of DKs in skin biopsy of JIA patients have been reported, larger studies of skin biopsy throughout the age spectrum in Still’s are warranted (18, 60). Third, the diagnostic potential of other tissue biopsy sites in Still’s should be explored. Lymph node biopsies may show reactive features such as paracortical hyperplasia as well as S100+ histiocyte proliferation, the latter of which can mimic a histiocyte disorder (61).

DKs in superficial epidermal layers are a common finding in skin biopsies of patients with AOSD. Although historically associated with the PPE, DKs can be seen in persistent rashes of AOSD with a variety of clinical lesions. In the correct clinical context, DKs are very specific for AOSD and should be included in future diagnostic and classification criteria.

## Materials and methods

### Study approval

Ethics approval for this minimal risk study was obtained from the University of British Columbia Clinical Ethics Research Board (H25-00959).

### Patient selection

We performed a retrospective review of medical records from the Coastal Rare Inflammatory Diseases Program based at Vancouver General Hospital and the Nova Scotia Health Research & Innovation Hub for patients diagnosed with AOSD who underwent a skin biopsy between 2016 and 2025. The Coastal Rare Diseases (CoRID) program is a Canadian program that serves patients with rare inflammatory blood diseases by providing specialist physicians with a second opinion from the senior author (L.Y.C. Chen) and a network of consultants. Patients over the age of 16 years with a diagnosis of AOSD by Yamaguchi criteria and a skin biopsy were included.

### Chart review

Demographic characteristics, clinical manifestations, and laboratory markers were collected. Skin biopsy reports were reviewed, and morphologic findings (epidermal changes, distribution and presence of DKs, interface changes, infiltration type, and mucin deposition) were recorded.

Cutaneous descriptions were taken from clinical notes. When available, descriptions documented by a dermatologist were preferentially used over descriptions from other specialists. Cutaneous findings were categorized as typical or atypical. Descriptors suggesting a typical rash were salmon-colored, evanescent, maculopapular, or transient. Atypical descriptions included any rash morphology outside of the former. However, this often included descriptors such as persistent, urticarial, papular, and pruritic.

### Dermatopathological assessment and histopathological definitions

Skin biopsy specimens were reviewed from original slides by certified dermatopathologists (D. Chen, I.M. Lano, S. Pasternak, and R.I. Crawford) at our institutions. Histological patterns and key findings were systematically documented. A DK was defined as a keratinocyte with abnormal condensation of the cytoplasmic keratin filaments, leading to intensely eosinophilic cytoplasmic staining. A necrotic keratinocyte was defined as one showing nuclear changes that indicate cell death, either karyolysis (nuclear loss), karyorrhexis (nuclear fragmentation), or nuclear pyknosis (extreme chromatin condensation). The necrotic keratinocyte can show a variety of cytoplasmic alterations (including, at times, dyskeratosis). Although necrotic keratinocytes can be seen in AOSD, the focus of this study was on DKs.

### Expert consensus and targeted literature review

An expert consensus list of dermatologists and dermatopathologists involved in this study was consulted on conditions in which DKs in superficial epidermal layers are seen. We supplemented this with a targeted literature search using the following search strategy: PubMed and Google Scholar were interrogated using search terms including “DKs,” “necrotic keratinocytes AND skin,” and “cutaneous histology AND keratinocytes” from January 1, 1990, to February 1, 2026. We searched the references of relevant manuscripts and conducted citation harvesting on Google Scholar for the most highly cited manuscripts.

### Online supplemental material

Baseline clinical and laboratory features of the patients are included in Table S1. An overview of the role of histological findings in diagnosing selected cytokine storm and autoinflammatory syndromes that overlap with Still’s disease is provided in Table S2. Some examples of the clinical skin lesions found in patients in this study are provided in Fig. S1.

## Supplementary Material

Table S1 shows baseline patient characteristics.

Table S2 shows histology in AOSD and other autoinflammatory/cytokine storm syndromes.

## Data Availability

Data are available upon reasonable request from the corresponding author.
