# Supplementary material for: Skin biopsy in adult-onset Still’s disease: A specific, rapid, and widely available diagnostic test
Source: J Hum Immun. 2026 Sep 18;2(6):e20260088. doi: 10.70962/jhi.20260088 (PMC13587882; doi:10.70962/jhi.20260088)
Supplement: Supplementary file 1 [file jhi_20260088_tables1.docx]

**Supplemental Table 1. Baseline Patient Characteristics**

| **#** | **Age/Sex** | **Clinical presentation** | **ANC**  (2-7.5 giga/L) | **CRP**  (<3.1 mg/L) | **Ferritin**  (<300 µg/L) | **sCD25**  (<852 U/L) | **cAOSD** | **Differential diagnosis at presentation** |
| --- | --- | --- | --- | --- | --- | --- | --- | --- |
| 1 | 62/F | Fv, Ln, Ph, Arth, Sm | 13 | 312 | 18192 | 1779 | Yes - HLH | Langerhan's histiocytosis, lymphoma, HLH |
| 2 | 36/M | Fv, Arth | 17.6 | 104.6 | >15000 | 3829 | Yes – ICU | AOSD, HLH, MAS |
| 3 | 48/F | Fv, Ln, Arth | 11.2 | 134 | 3832 | 3078 | no | AOSD, HLH |
| 4 | 44/F | Fv, Ln, Ph, Arth | 11.5 | 117 | 4365 | 1507 | no | AOSD, HLH, LPD, MAS |
| 5 | 19/F | Fv, Ln, Ph, Arth, Sm | 9.7 | 300 | 5411 | 3124 | no | AOSD, HLH, LPD |
| 6 | 34/M | Fv, Ln, Arth | 33 | 316 | 11,443 | 1168 | no | AOSD, HES |
| 7 | 50/F | Fv*, Ph, Arth | 7.5 | 88.9 | 790 | ND | no | Infection, malignancy |
| 8 | 32/F | Fv, Ph, Arth | 8.4 | 53.7 | 14,733 | ND | no | Autoimmune vs malignancy |
| 9 | 36/F | Fv*, Ph, Arth | 6.9 | 63.8 | 1498 | ND | no | inflammatory arthritis |
| 10 | 43/M | Fv, Ph, Arth, liver failure, MAS, DIC | 14.8 | 240 | 85020 | 5425 | Yes - HLH | Infectious or autoimmune hepatitis, HLH, malignancy |
| 11 | 23/F | Fv, Ph, Arth, myocarditis, cardiogenic shock | 4.5 | 228 | 2272 | 229 | Yes - ICU | AOSD, HLH, Kawasaki, MISC |
| 12 | 40/F | Fv, Ln, Arth | 8.8 | 339 | 4116 | ND | no | Viral urticaria, AOSD, lymphoma |
| 13 | 66/M | Fv, Ln, Ph, Arth, Sm | 36.9 | 363 | 2389 | ND | no | AOSD, HLH, MAS, malignancy, vasculitis, virus, drug reaction |
| 14 | 57/F | Fv, Ln, Ph, Arth | 12.3 | 184 | 36,323 | ND | no | AOSD, HLH, lymphoma |
| 15 | 34/F | Fv, Arth | 18.1 | 296 | 54,773 | ND | no | AOSD, HLH/MAS, hematologic malignancy |
| 16 | 37/F | Fv, Ph, Arth, | 24.1 | 279 | 29,434 | 1636 | Yes - HLH | Autoimmune process, malignancy |

ANC=absolute neutrophil count, Arth = arthralgia, cAOSD = catastrophic Adult onset Still’s Disease as defined by Wahbi et al.^6^ DIC = disseminated intravascular coagulation, Fv = Fever > 39C (* subjective fevers), Hm = hepatomegaly, LN = lymphadenopathy, MAS = macrophage activation syndrome, ND = not done, Ph = pharyngitis, Ple = pleural effusion, Sm = splenomegaly
