# Supplementary material for: Skin biopsy in adult-onset Still’s disease: A specific, rapid, and widely available diagnostic test
Source: J Hum Immun. 2026 Sep 18;2(6):e20260088. doi: 10.70962/jhi.20260088 (PMC13587882; doi:10.70962/jhi.20260088)
Supplement: Supplementary file 2 [file jhi_20260088_tables2.docx]

**Supplemental Table 2: role of histology in selected cytokine storm/autoinflammatory diseases**

| **Condition** | **Epidemiology*** | **Biopsy** | **Genetics** | **Notes** |
| --- | --- | --- | --- | --- |
| Schnitzler sydnrome | Adult onset | Skin – neutrophilic dermatosis | No causative genes identified | 90% have a monoclonal IgM paraprotein on SPEP |
| Hemophagocytic lymphohistiocytosis (HLH) | Primary – children; Secondary - adults | Bone marrow – hemophagocytosis – non specific | Many genes in primary HLH [e.g. PRF1, UnCD13D, STX11, XIAP, etc]  Other important emerging genes in IEI implicated in HLH such as HAVCR2, GATA2, etc. | Hemophagocytosis is a diagnostic criterion but is non specific – often seen in TAFRO, stills |
| VEXAS (vacuoles, E1 enzyme, X chromosome, autoinflammatory, somatic) syndrome | Men > 50 years | Bone marrow – abundant vacuoles in erythroid and myeloid precursors | UBA1 | Vacuolization quite sensitive and specific*** |
| TAFRO (thrombocytopenia, anasarca, fibrosis, renal dysfunction, organomegaly) syndrome | Men 30-50 more common  More common in Asians | Lymph node – Castleman disease changes, typically hyaline vascular** | No causative genes identified | Lymph nodes are often small volume, difficult to biopsy |
| Still’s disease | Juvenile idiopathic arthritis – children  adult onset Still’s - adults | **Skin** - dyskeratosis in superficial epidermal layers  **Lymph node** – reactive changes including abundant S100 positive histiocytes  **Other tissues** - Hemophagocytosis and lymphohistiocytic infiltrate | No causative genes identfied | Skin biopsy is easy to obtain, easy to interpret |

**25% of TAFRO has no LAD, therefore no CD

*** other causes include Copper, zinc deficiency, MDS, alcohol, but not as many vacuoles are seen in these conditions
